# Regenerated hair cells in the neonatal cochlea are innervated and the majority co-express markers of both inner and outer hair cells

**DOI:** 10.3389/fncel.2022.841864

**Published:** 2022-09-16

**Authors:** Mitchell L. Heuermann, Sophia Matos, Deborah Hamilton, Brandon C. Cox

**Affiliations:** ^1^Department of Otolaryngology, Southern Illinois University School of Medicine, Springfield, IL, United States; ^2^Department of Pharmacology, Southern Illinois University School of Medicine, Springfield, IL, United States

**Keywords:** hair cell regeneration, inner hair cells, outer hair cells, hair cell development, fate mapping, oncomodulin, VGlut3

## Abstract

After a damaging insult, hair cells can spontaneously regenerate from cochlear supporting cells within the first week of life. While the regenerated cells express several markers of immature hair cells and have stereocilia bundles, their capacity to differentiate into inner or outer hair cells, and ability to form new synaptic connections has not been well-described. In addition, while multiple supporting cell subtypes have been implicated as the source of the regenerated hair cells, it is unclear if certain subtypes have a greater propensity to form one hair cell type over another. To investigate this, we used two CreER mouse models to fate-map either the supporting cells located near the inner hair cells (inner phalangeal and border cells) or outer hair cells (Deiters’, inner pillar, and outer pillar cells) along with immunostaining for markers that specify the two hair cell types. We found that supporting cells fate-mapped by both CreER lines responded early to hair cell damage by expressing Atoh1, and are capable of producing regenerated hair cells that express terminal differentiation markers of both inner and outer hair cells. The majority of regenerated hair cells were innervated by neuronal fibers and contained synapses. Unexpectedly, we also found that the majority of the laterally positioned regenerated hair cells aberrantly expressed both the outer hair cell gene, oncomodulin, and the inner hair cell gene, vesicular glutamate transporter 3 (VGlut3). While this work demonstrates that regenerated cells can express markers of both inner and outer hair cells after damage, VGlut3 expression appears to lack the tight control present during embryogenesis, which leads to its inappropriate expression in regenerated cells.

## Introduction

The organ of Corti, located within the cochlea of the inner ear, contains the intricate anatomy required for hearing. It is comprised of three rows of outer hair cells, one row of inner hair cells, various supporting cell subtypes, and innervating fibers of spiral ganglion neurons. Whereas inner hair cells act as mechanoelectric transducers that encode sound waves into spiral ganglion neuronal action potentials, outer hair cells amplify sound input to increase frequency specificity and the dynamic range of hearing (reviewed in Dallos, 2008). Both types of hair cells form afferent synaptic connections with spiral ganglion neurons, although these differ in function and number. Each inner hair cell forms synapses with multiple type I afferent spiral ganglion neurons (Liberman, 1980; Bohne et al., 1982; Stamataki et al., 2006), although each type I afferent will synapse with only one inner hair cell (Spoendlin, 1969; Kiang et al., 1982; Liberman, 1982). Type I neurons are responsible for carrying action potentials toward the ascending auditory pathway (Reijntjes and Pyott, 2016). Type II spiral ganglion neurons form synapses with outer hair cells (Huang et al., 2012; Martinez-Monedero et al., 2016; Elliott et al., 2021). Although their function is less understood, they are proposed to have a nociceptive function in response to mechanical or noise trauma (Weisz et al., 2009; Flores et al., 2015; Liu et al., 2015).

Hearing loss is multifactorial and can include insults from noise exposure, aging, genetic disorders, and ototoxic drugs. It is among the most prevalent diseases, with an estimated 1.5 billion individuals affected (Gbd 2019 Hearing Loss Collaborators, 2021). Because of this, there has been a keen interest in treating or reversing hearing loss. As most forms of hearing damage culminate with the loss of hair cells, much of this research has focused on regenerating new hair cells. While the mammalian cochlea can spontaneously regenerate its hair cells at neonatal ages (White et al., 2006; Bramhall et al., 2014; Cox et al., 2014; Hu et al., 2016), no regeneration occurs after the first postnatal week (Oesterle et al., 2008; Cox et al., 2014; Maass et al., 2015).

A number of studies have demonstrated that supporting cells act as the progenitor cells that form new hair cells during regeneration using two mechanisms. First, with direct transdifferentiation, a supporting cell changes fate to a hair cell without leaving quiescence. Second, with mitotic regeneration, a supporting cell divides, and one or both daughter cells differentiate into hair cells (Rubel et al., 2013; Bramhall et al., 2014; Cox et al., 2014). At least eight subtypes of supporting cells have been identified in the mammalian cochlea, including Claudius cells, Hensen’s cells, Deiters’ cells, inner and outer pillar cells, inner phalangeal cells, border cells and greater epithelial ridge cells (Raphael and Altschuler, 2003; Jahan et al., 2015). Available evidence suggest that multiple supporting cell subtypes are capable of regenerating hair cells, although it is debated whether certain subtypes have a greater propensity over others (Bramhall et al., 2014; McGovern et al., 2019). In addition, two studies have shown that regenerated hair cells can differentiate into an outer hair cell fate (Bramhall et al., 2014; Cox et al., 2014); yet it is unknown how many reach terminal differentiation and whether inner hair cells can also be replaced. Furthermore, while regenerated hair cells formed in non-mammalian vertebrates contain synapses and are innervated (Duckert and Rubel, 1990; Ryals and Westbrook, 1994), the capacity for innervation in regenerated hair cells in mammals has not been explored. Each of these questions is of vital importance to understand the extent to which spontaneous regeneration drives cochlear repair.

Here, we investigated the terminal differentiation and innervation of regenerated hair cells in the neonatal mouse cochlea using CreER mouse models to fate-map either the supporting cells surrounding inner hair cells (inner phalangeal and border cells) or those that neighbor outer hair cells (Deiters’, inner pillar, and outer pillar cells). We found that supporting cells fate-mapped by both CreER lines were capable of producing regenerated hair cells that expressed terminal differentiation markers of inner and outer hair cells, the majority of which were innervated by neuronal fibers and contained synapses. In addition, the majority of regenerated cells in the lateral compartment (where outer hair cells are normally located) showed aberrant expression of both oncomodulin (an outer hair cell marker) and vesicular glutamate transporter 3 (VGlut3) (an inner hair cell marker).

## Results

**FIGURE 1:**
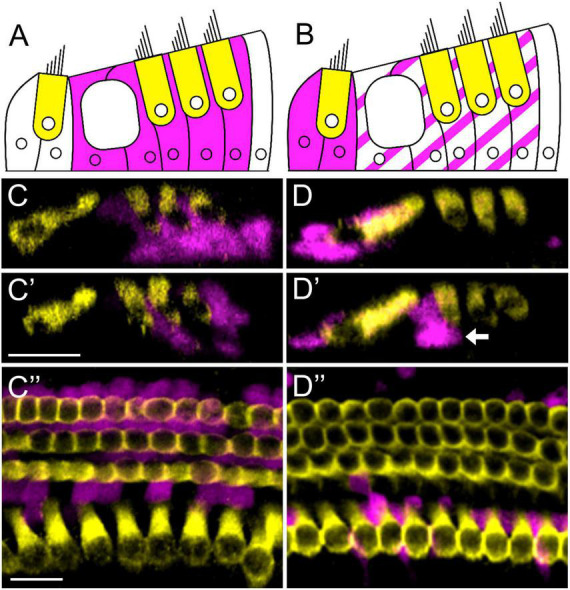
*Prox1^CreERT2^* and *Plp-CreER*^T^** label different groups of cochlear supporting cell subtypes. **(A,B)** Illustration of a cochlear cross-section with one row of inner and three rows of outer hair cells (yellow) and tdTomato-positive (magenta) supporting cells labeled by *Prox1^CreERT2^* **(A)** or *Plp-CreER*^T^** **(B)**. **(C–D”)** Representative images from the apical turn of *Prox1*^CreERT2^*:Rosa26*^tdTomato^** **(C–C”)** and *Plp-CreER*^T^*:Rosa26*^tdTomato^** **(D–D”)** mice at P7. All mice were injected with tamoxifen at P0 to induce tdTomato expression in various groups of supporting cells. Samples were immunostained with anti-Myo7a (yellow) to label all hair cells, and tdTomato was detected using endogenous fluorescence (magenta). *Prox1^CreERT2^* labels primarily pillar and Deiters’ cells **(A,C–C”)**, whereas *Plp-CreER*^T^** labels primarily inner phalangeal and border cells (solid magenta in **B**), along with a small number of pillar and Deiters’ cells (striped magenta in **B**) **(B,D–D”)**. White arrow **(D’)** indicates an outer pillar cell labeled by *Plp-CreER*^T^**. Panels **(C–D’)** are optical cross-sections and panels **(C”–D”)** are top-down views. All scale bars: 20 μm.

To investigate whether different supporting cells subtypes can produce regenerated cells that differentiate into inner or outer hair cell fates, we used two CreER lines (*Prox1^CreERT2^* and *Plp-CreER*^T^**) that were paired with the reporter, *Rosa26^CAG–loxP–stop–loxP–tdTomato^* (*Rosa26^tdTomato^*) for fate-mapping. When injected with tamoxifen at postnatal day (P) 0, *Prox1^CreERT2^:Rosa26*^tdTomato^** mice have previously been shown to selectively label inner pillar (∼33%), outer pillar (∼78%), and Deiters’ (∼78%) cells (Figures 1A,C–C”; Yu et al., 2010; Liu et al., 2012; McGovern et al., 2017), while *Plp-CreER*^T^*:Rosa26*^tdTomato^** mice primarily labeled inner phalangeal and border cells (∼73%), as well as a small number of inner pillar (∼7%), outer pillar (∼9%), and Deiters’ cells (∼11%) (Figures 1B,D–D”; Gómez-Casati et al., 2010; McGovern et al., 2017). These two lines (*Prox1^CreERT2^:Rosa26*^tdTomato^** and *Plp-CreER*^T^*:Rosa26*^tdTomato^**) were then crossed with *Pou4f3^DTR^* mice which express the human diphtheria toxin receptor (DTR) specifically in hair cells using the *Pou4f3* promoter. Administration of diphtheria toxin (DT) to mice carrying the *Pou4f3^DTR^* allele then leads to selective hair cell death. *Prox1^CreERT2^:Rosa26*^tdTomato^*:Pou4f3*^DTR^** and *Plp-CreER*^T^*:Rosa26*^tdTomato^**:*Pou4f3^DTR^* mice (referred to hereafter as *DTR:Prox1^CreERT2^* and *DTR:Plp-CreER*^T^**) served as the experimental groups, while their littermates lacking the *Pou4f3^DTR^* allele (and therefore not susceptible to DT-induced hair cell damage) were used as controls. All mice were injected with tamoxifen (3 mg/40 g, intraperitoneally) on P0, thereby inducing tdTomato expression in the specific supporting cell subtypes described above and with DT (6.25 ng/g, intramuscularly) on P1 to cause selective death of the hair cells and induce subsequent hair cell regeneration, as previously described (Cox et al., 2014). Cochleae were harvested on P3-P10 and the entire organ of Corti was analyzed from apex to base using immunostaining and confocal microscopy. To identify regenerated cells that differentiated toward either an inner or outer hair cell fate, we labeled all hair cells using anti-myosin VIIA (Myo7A) antibodies and used additional antibodies that were specific to inner or outer hair cells. Regenerated hair cells (Myo7a-positive) were identified based on tdTomato expression, which was controlled by the supporting cell-specific CreER lines. In *Plp-CreER*^T^** control samples at P7, we observed an average of 1.6 ± 0.8 tdTomato-positive outer hair cells and 0.4 ± 0.4 tdTomato-positive inner hair cells (*n* = 5), whereas *Prox1^CreERT2^* controls had an average of 13.4 ± 1.7 tdTomato-positive outer hair cells and no tdTomato-positive inner hair cells (*n* = 7). All tdTomato-positive hair cells in control samples were located almost exclusively at apical-most portion of each cochlea. Similar to previous reports (Cox et al., 2014; McGovern et al., 2019), the vast majority of the regenerate HCs (tdTomato-positive/Myo7a-positive cells) were located in the apical turn.

### A large number of supporting cells respond to hair cell damage by expressing Atoh1, but only a fraction of these cells mature to express myosin VIIA

**FIGURE 2:**
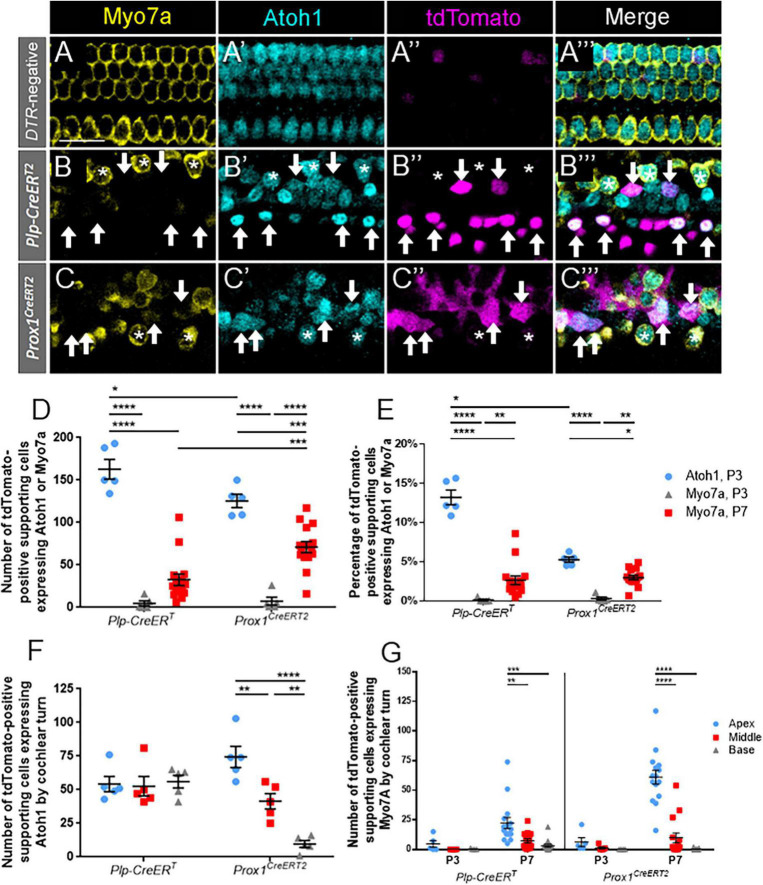
A significant proportion of Atoh1-positive supporting cells at P3 do not mature to Myo7a-positive regenerated hair cells by P7. **(A–C”’)** Representative confocal slice images from the apical turn of *Atoh1^GFP/+^* **(A–A”’)**, *Plp-CreER*^T^*:Rosa26*^tdTomato^*:Pou4f3*^DTR^*:Atoh1*^GFP/^**^+^ **(B–B”’)** and *Prox1*^CreERT2^*:Rosa26*^tdTomato^*:Pou4f3*^DTR^*:Atoh1*^GFP/^**^+^ **(C–C”’)** mice at P3. All mice were injected with tamoxifen at P0 to induce tdTomato expression in various groups of supporting cells and with DT at P1 to induce hair cell damage. Samples were immunostained with anti-Myo7a antibodies (yellow) to label all hair cells and anti-GFP antibodies (blue) to detect Atoh1 expression as an early marker of hair cell conversion. tdTomato was detected using endogenous fluorescence (magenta). Arrows indicate Atoh1-positive/Myo7a-negative/tdTomato -positive fate-mapped supporting cells. Asterisks indicate Myo7a-positive/tdTomato-negative cells that are hair cells which survived the DT-mediated damage. Note that in controls all Atoh1-postive cells also expressed Myo7a. Scale bars: 25 μm. **(D)** The supporting cell populations fate-mapped by *Plp-CreER*^T^** produced significantly more cells expressing Atoh1 than those fate-mapped by *Prox1^CreERT2^*. A significant number of the hair cell progenitors from both populations of supporting cells did not mature to Myo7a-positive regenerated hair cells by P7, although the number of Myo7a-positive regenerated hair cells present at P7 was significantly greater for in the *Prox1^CreERT2^* group compared the *Plp-CreER*^T^** group. **(E)** Data from panel **(D)** normalized to the total number of Tomato-positive supporting cells in each mouse model. **(F)** When stratified by cochlear turn at P3, there were similar numbers of Atoh1-positive, *Plp-CreER*^T^**-positive supporting cells in each turn, but there was a decreasing gradient of Atoh1-positive, *Prox1^CreERT2^*-positive supporting cells from apex to base. **(G)** Supporting cell populations in both CreER lines produced significantly more regenerated hair cells in the apex compared to the middle and basal turns at P7, but there were no differences across cochlear turns at P3. Data are presented as mean ± SEM for *n* = 5–15. *p < 0.05; **p < 0.01; ***p < 0.001; ****p < 0.0001 determined using two-way ANOVA with a Sidak’s *post-hoc* test.

We first sought to compare the response to hair cell damage among supporting cell subtypes using expression of *Atoh1*, the first known gene to be turned on in a cell differentiating into a hair cell fate (Bermingham et al., 1999). *DTR:Prox1^CreERT2^* and *DTR:Plp-CreER*^T^** mice were bred with *Atoh1^GFP/+^* mice, which is a knock-in reporter line that creates an ATOH1-GFP fusion protein (Rose et al., 2009). Both groups were treated with tamoxifen on P0 and DT on P1, and temporal bones were collected on P3. Endogenous GFP fluorescence was augmented with anti-GFP antibodies. In control *Prox1^CreERT2^* and *Plp-CreER*^T^** samples at P3, Atoh1 expression was seen exclusively in Myo7a-positive hair cells, with only three Atoh1-positive supporting cells observed among three control cochleae (Figures 2A–A”’). Next, we examined experimental mice that had hair cell damage to quantify tdTomato-positive supporting cells that expressed ATOH1-GFP, but lacked the more mature hair cell marker, Myo7a (Figures 2B–C”’). At P3, there was a larger number of supporting cells that expressed ATOH1-GFP after hair cell damage in *DTR:Plp-CreER*^T^** mice (162.8 ± 11.7, *n* = 5) compared to *DTR:Prox1^CreERT2^* mice (125.4 ± 7.8, *n* = 5, *p* = 0.0031 determined using a two-way ANOVA followed by a Sidak’s *post-hoc* test) (Figure 2D). When converted to a percentage (using the total number of Tomato-positive supporting cells from each CreER line), only 13.2 ± 0.9% of the Cre-positive supporting cells in *DTR:Plp-CreER*^T^** mice and 5.3 ± 0.3% of the Cre-positive supporting cells in *DTR:Prox1^CreERT2^* mice expressed Atoh1-GFP (*n* = 5; Figure 2E). However comparing across cochlear turns, *DTR:Prox1^CreERT2^* mice showed a decreasing gradient of Atoh1-GFP-positive/Tomato-positive cells from apex to base (74.4 ± 7.9 in apex, 41.4 ± 5.8 in middle, and 9.6 ± 2.6 in base, *n* = 5, *p* = 0.0051 determined using a two-way ANOVA followed by a Sidak’s *post-hoc* test), but there was no difference among turns in the *DTR:Plp-CreER*^T^** mice (Figure 2E). This may suggest that supporting cells in the medial compartment remain plastic for a longer time period than the lateral supporting cells. Also at P3, a similar, but very small number of cells co-expressed tdTomato and Myo7a in both CreER lines (4.8 ± 3.1 for *Plp-CreER*^T^** vs. 7.4 ± 4.7 for *Prox1^CreERT^*, *n* = 5; Figure 2D), which is less than 0.5% of the total Tomato-positive supporting cells in each CreER line (*n* = 5; Figure 2E).

We then determined the number of tdTomato-positive supporting cells that progressed to express Myo7a at P7 (Figure 2D). Numbers of regenerating, Myo7a-positive/Tomato-positive hair cells fate-mapped by *Prox1^CreERT2^* were two-fold greater than those fate-mapped by *Plp-CreER*^T^** at P7 (71.1 ± 6.5 vs. 32.7 ± 6.8, *n* = 15, *p* < 0.0001 determined using a two-way ANOVA followed by a Sidak’s *post-hoc* test). When converted to a percentage, this accounts for ∼2.5–3% of the Tomato-positive supporting cells in each CreER line (*n* = 5; Figure 2E). When comparing across cochlear turns, both *DTR:Plp-CreER*^T^** and *DTR:Prox1^CreERT2^* mice showed a decreasing gradient of Myo7a-positive/Tomato-positive cells from apex to base (*DTR:Plp-CreER*^T^**: 22.3 ± 4.7 in apex, 7.3 ± 1.7 in middle, and 3.1 ± 1.2 in base, *n* = 15, *p* = 0.003; *DTR:Prox1^CreERT2^*: 61.1 ± 5.9 in apex, 9.8 ± 4.1 in middle, and 0.2 ± 0.1 in base; *n* = 15, *p* < 0.0001 determined using a two-way ANOVA followed by a Sidak’s *post-hoc* test) (Figure 2G). Compared to the number of ATOH1-GFP-positive supporting cells at P3, cells expressing tdTomato and Myo7a at P7 were 2–4 fold less in both models (162.8 ± 11.7 ATOH1-GFP/tdTomato, *n* = 5 vs. 32.7 ± 6.8 Myo7a/tdTomato, *n* = 15 for *Plp-CreER*^T^**, *p* < 0.0001, and 125.4 ± 7.8 ATOH1-GFP/tdTomato, *n* = 5 vs. 71.1 ± 6.5 Myo7a/tdTomato, *n* = 15 for *Prox1^CreERT2^*, *p* < 0.0001 determined using a two-way ANOVA followed by a Sidak’s *post-hoc* test) (Figure 2D). This suggests that a significant number of supporting cells respond to hair cell damage by expressing Atoh1, but they have not yet completed the transdifferentiation process by P7.

### Oncomodulin expression is seen in a similar number and percentage of regenerated hair cells derived from all supporting cell subtypes

**FIGURE 3:**
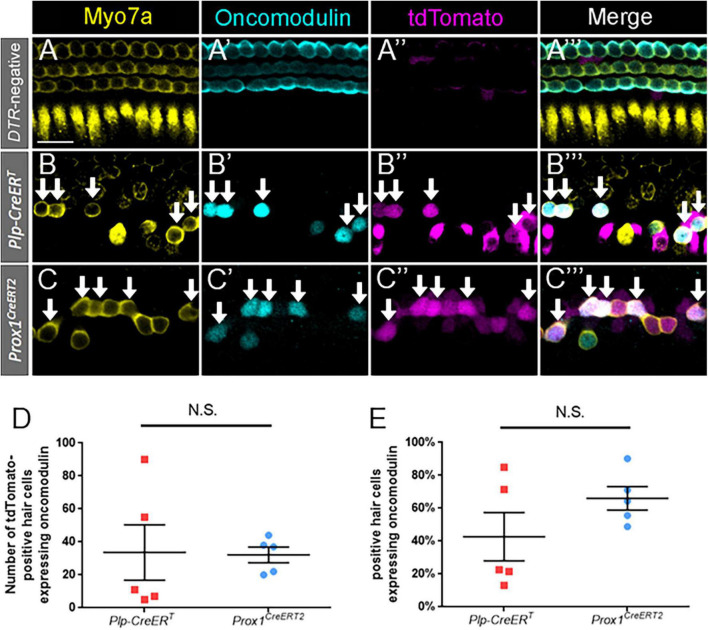
Oncomodulin is expressed in a similar number and percentage of regenerated hair cells across supporting cell subtypes. **(A–C”’)** Representative confocal slice images from the middle turn of *Pou4f3^DTR^*-negative **(A–A”’)** and apical turns of *Plp-CreER*^T^*:Rosa26*^tdTomato^*:Pou4f3*^DTR^** **(B–B”’)** and *Prox1*^CreERT2^*:Rosa26*^tdTomato^*:Pou4f3*^DTR^** **(C–C”’)** mice at P7–P8 that were injected with tamoxifen at P0 to induce tdTomato expression in the two supporting cell subpopulations and with DT at P1 to induce hair cell damage. Samples were immunostained with anti-Myo7a antibodies (yellow) to label all hair cells and anti-oncomodulin antibodies (blue) to label immature outer hair cells. tdTomato was detected using endogenous fluorescence (magenta). Arrows indicate Myo7a-positive/oncomodulin-positive/tdTomato-positive cells that are regenerated, fate-mapped outer hair cells. Scale bar: 25 μm. **(D)** There was no significant difference in the total number of regenerated hair cells expressing oncomodulin between the supporting cell populations targeted by the two CreER lines. **(E)** When normalized to the total number of tdTomato-positive/Myo7a-positive cells, there was no significant difference in the percentage of oncomodulin-positive regenerated hair cells between the supporting cell populations targeted by the two CreER lines. Significance was determined using an unpaired Student’s *t*-test. Data are presented as mean ± SEM; *n* = 5. N.S., not significant.

To identify immature outer hair cells, cochlear sections were costained with oncomodulin (also called parvalbumin-β) and Myo7a. Oncomodulin is a calcium binding protein that has been previously shown to be selectively expressed in immature outer hair cells beginning as early as P2 (Hackney, 2005; Simmons et al., 2010; Tong et al., 2016). In control *Prox1^CreERT2^* and *Plp-CreER*^T^** samples at P7, oncomodulin expression was specific to outer hair cells, with only one oncomodulin-positive inner hair cell observed among three control cochleae, and no expression detected in supporting cells (Figures 3A–A”’). Robust oncomodulin expression was also seen at P7 in regenerated hair cells (tdTomato-positive/Myo7a-positive) fate-mapped by both CreER lines (Figures 3B–C”’). There were no Tomato-positive/Myo7a-negative cells that expressed oncomodulin in any of the control or experimental samples. Quantification of total numbers of oncomodulin-positive regenerated hair cells (tdTomato-positive/Myo7a-positive) were similar for both CreER lines (Figure 3D; 32.2 ± 4.7 for *DTR:Prox1^CreERT2^* vs. 33.6 ± 16.9 for *DTR:Plp-CreER*^T^**, *n* = 5, determined using an unpaired Student’s *t*-test), and remained similar when the counts were normalized to the total number of regenerated hair cells (tdTomato-positive/Myo7a-positive) in each sample (Figure 3E; 66.0 ± 7.1% for *DTR:Prox1^CreERT2^* vs. 42.7 ± 14.7% for *DTR:Plp-CreER*^T^**, *n* = 5, determined using an unpaired Student’s *t*-test). This suggests that both subsets of supporting cells have an equivalent capacity to differentiate toward an outer hair cell fate.

### Prestin expression increases with age and becomes proportionally higher in regenerated hair cells derived from lateral compartment supporting cells

To identify terminally differentiated outer hair cells, cochlear sections were costained with prestin and Myo7a. Prestin, the cytoplasmic motor protein important for the cochlear amplifier function, has been previously shown to be a reliable marker for mature outer hair cells, and it is not expressed in supporting cells or inner hair cells (Liberman et al., 2002; Fang et al., 2012; Bramhall et al., 2014; Cox et al., 2014). Previous work demonstrated that some spontaneously regenerated hair cells in the neonatal cochlea can express prestin (Bramhall et al., 2014; Cox et al., 2014).

**FIGURE 4:**
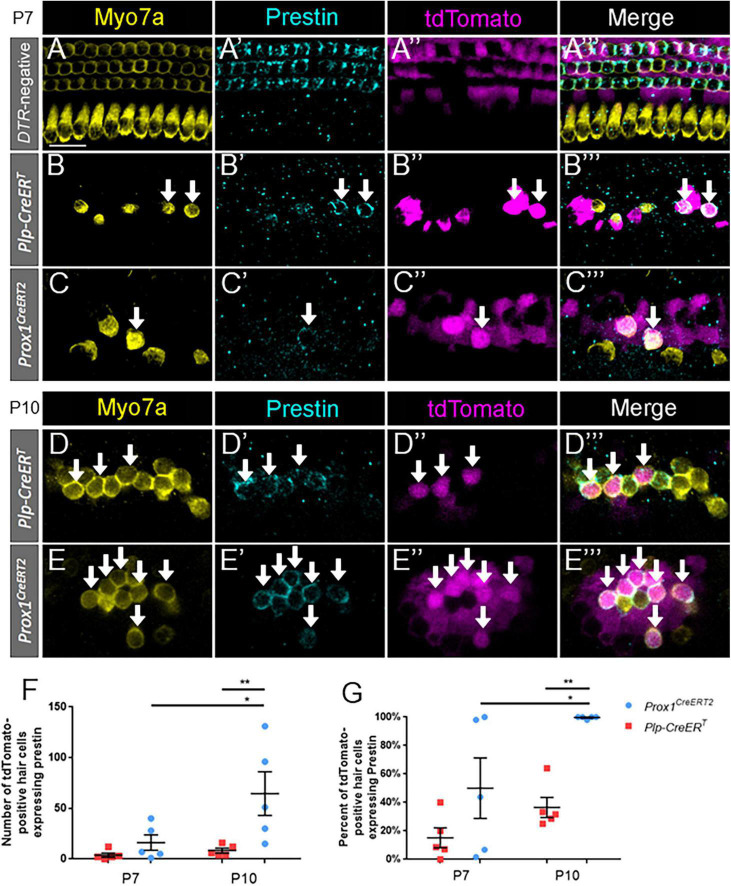
Prestin expression in regenerated hair cells increases with age and is proportionally higher in pillar and Deiters’ cells. **(A–E”’)** Representative confocal slice images from the middle turn of *Pou4f3^DTR^*-negative **(A–A”’)** and from the apical turn of *Plp-CreER*^T^*:Rosa26*^tdTomato^*:Pou4f3*^DTR^** **(B–B”’,D–D”’)** and *Prox1*^CreERT2^*:Rosa26*^tdTomato^*:Pou4f3*^DTR^** **(C–C”’,E–E”’)** mice at P7–P8 **(A–C”’)** and P10 **(D–E”’)** that were injected with tamoxifen at P0 to induce tdTomato expression in the two supporting cell subpopulations and with DT at P1 to induce hair cell damage. Samples were immunostained with anti-Myo7a antibodies (yellow) to label hair cells and anti-prestin antibodies (blue) to label mature outer hair cells. tdTomato was detected using endogenous fluorescence (magenta). Arrows indicate Myo7a-positive/prestin-positive/tdTomato-positive cells that are regenerated, fate-mapped mature outer hair cells. Scale bars: 25 μm. **(F)** A significantly greater number of prestin-positive regenerated hair cells were seen in the supporting cells located in the lateral compartment and fate-mapped by *Prox1^CreERT2^* between P7 and P10 and between the supporting cell populations targeted by the two CreER lines at P10. **(G)** Data from panel **(F)** normalized to the total number of Tomato-positive/Myo7a-positive cells in each mouse model. *p < 0.05; **p < 0.01, determined using two-way ANOVA with a Sidak’s *post-hoc* test. Data are presented as mean ± SEM; *n* = 5. N.S., not significant.

Like oncomodulin, prestin was specifically expressed in outer hair cells of control *Prox1^CreERT2^* and *Plp-CreER*^T^** samples at P7, with no prestin-positive inner hair cells or supporting cells seen among the three control cochleae (Figures 4A–A”’). Similar to oncomodulin, prestin was also expressed in regenerated hair cells (tdTomato-positive/Myo7a-positive) derived from both subsets of supporting cells. However, at P7, prestin expression appeared faint (Figures 4B–C”’) and there were low numbers and percentages of prestin-positive regenerated hair cells (tdTomato-positive/Myo7a-positive) (Figures 4F,G). Therefore, we also examined samples at P10 (Figures 4D–E”’), where prestin staining was stronger (Figures 4D’,E’ vs. Figures 4B’,C’). Regenerated hair cells derived from *Prox1^CreERT2^*-labeled supporting cells (in the lateral compartment) showed a significantly higher number of prestin-positive hair cells at P10 compared to P7 (64.6 ± 21.5 vs. 16.2 ± 7.6, *n* = 5, *p* = 0.0130 determined using a two-way ANOVA followed by a Sidak’s *post-hoc* test), but there was no difference in the number of cells expressing prestin between P7 and P10 in the *DTR:Plp-CreER*^T^** mice (Figure 4F). At P10, there were also more prestin-positive regenerated hair cells (tdTomato-positive/Myo7a-positive) in *DTR:Prox1^CreERT2^* samples than *DTR:Plp-CreER*^T^** samples (64.6 ± 21.5 vs. 8.4 ± 2.4, *n* = 5, *p* = 0.0065 determined using a two-way ANOVA followed by a Sidak’s *post-hoc* test) (Figure 4F). Likewise, when the data were converted to a percentage [to normalize it to the total number of regenerated hair cells (tdTomato-positive/Myo7a-positive)], regenerated hair cells derived from *Prox1^CreERT2^*-labeled supporting cells (in the lateral compartment) showed a significantly higher percentage of prestin-positive hair cells at P10 compared to P7 (99.5 ± 0.4% vs. 50.0 ± 21.2%, *n* = 5, *p* = 0.0325 determined using a two-way ANOVA followed by a Sidak’s *post-hoc* test), and compared to those derived from *Plp-CreER*^T^**-labeled supporting cells at P10 (99.5 ± 0.4% vs. 36.5 ± 7.0%, *n* = 5, *p* = 0.0031 determined using a two-way ANOVA followed by a Sidak’s *post-hoc* test) (Figure 4G). There were no Tomato-positive/Myo7a-negative cells that expressed prestin in any of the control or experimental samples. These data suggest that similar to normal development, it takes several days after Myo7a expression for prestin to be expressed in regenerated outer hair cells, and prestin-positive regenerated hair cells are preferentially produced by supporting cells originating in the lateral compartment, which neighbor the endogenous outer hair cells.

### VGlut3 is expressed in hair cells regenerated from all supporting cell subtypes

**FIGURE 5:**
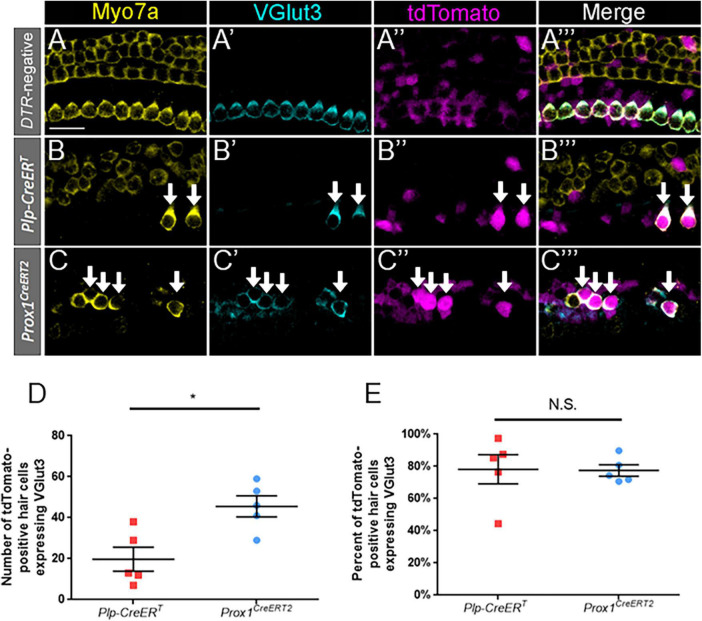
VGlut3 is expressed in a similar proportion of regenerated hair cells across supporting cell subtypes. **(A–C”’)** Representative confocal slice images from the apical turn of *Pou4f3^DTR^*-negative **(A–A”’)**, middle turn of *Plp-CreER*^T^*:Rosa26*^tdTomato^*:Pou4f3*^DTR^** **(B–B”’)**, and apical turn of *Prox1*^CreERT2^*:Rosa26*^tdTomato^*:Pou4f3*^DTR^** **(C–C”’)** mice at P7–P8 that were injected with tamoxifen at P0 to induce tdTomato expression in the two supporting cell subpopulations and with DT at P1 to induce hair cell damage. Samples were immunostained with anti-Myo7a antibodies (yellow) to label hair cells and anti-VGlut3 antibodies (blue) to label mature inner hair cells. tdTomato was detected using endogenous fluorescence (magenta). Arrows indicate Myo7a-positive/VGlut3-positive/tdTomato-positive cells that are regenerated, fate-mapped mature inner hair cells. Scale bars: 25 μm. **(D)** Significantly more regenerated hair cells derived from supporting cells in the lateral compartment and fate-mapped by *Prox1^CreERT2^* expressed VGlut3. **(E)** When normalized to the total number of tdTomato-positive/Myo7a-positive cells, there was no significant difference in the percentage of VGlut3-positive regenerated hair cells between the supporting cell populations targeted by the two CreER lines. *p < 0.05, determined using an unpaired Student’s *t*-test. Data are presented as mean ± SEM; *n* = 5. N.S., not significant.

To identify terminally differentiated inner hair cells, cochlear sections were costained with VGlut3 and Myo7a. VGlut3 is responsible for transport of cytoplasmic glutamate into presynaptic vesicles of inner hair cells, and is not normally expressed in outer hair cells or supporting cells (Seal et al., 2008; Liu et al., 2014; Strenzke et al., 2016). We confirmed this expression pattern of VGlut3 in our control *Prox1^CreERT2^* and *Plp-CreER*^T^** samples at P7 (Figures 5A–A”’); no VGlut3-positive outer hair cells or supporting cells were seen among three control cochleae. Like the outer hair cell markers, VGlut3 expression was also seen in regenerated hair cells (tdTomato-positive/Myo7a-positive) fate-mapped by both *Prox1^CreERT2^* and *Plp-CreER*^T^** at P7 (Figures 5B–C”’). There were no Tomato-positive/Myo7a-negative cells that expressed VGlut3 in any of the control or experimental samples. While regenerated hair cells (tdTomato-positive/Myo7a-positive) of *Prox1^CreERT2^* supporting cell origin had a greater number of VGlut3-positive cells than those of *Plp-CreER*^T^** origin (45.6 ± 5.2 vs. 19.8 ± 5.9, *n* = 5, *p* = 0.0108 determined using an unpaired Student’s *t*-test) (Figure 5D), when normalized to the total number of regenerated hair cells (tdTomato-positive/Myo7a-positive), there was a similar percentage of VGlut3-positive cells in both CreER lines (77.5 ± 3.5% and 78.2 ± 9.1%, *n* = 5) (Figure 5E). This suggests that both subsets of supporting cells have an equivalent capacity to differentiate toward an inner hair cell fate.

### Some regenerated hair cells aberrantly express inner and outer hair cell markers simultaneously

Given the high percentage of regenerated cells (tdTomato-positive/Myo7a-positive) that expressed oncomodulin and VGlut3 in both of the CreER lines (Figures 3E, 5E), we hypothesized that some cells expressed both of these genes. To test this, *DTR:Prox1^CreERT2^* and *DTR:Plp-CreER*^T^** cochlear samples were stained with both oncomodulin and VGlut3 at P7. Regenerated hair cells were identified by tdTomato expression combined with expression of either or both oncomodulin and VGlut3.

**FIGURE 6:**
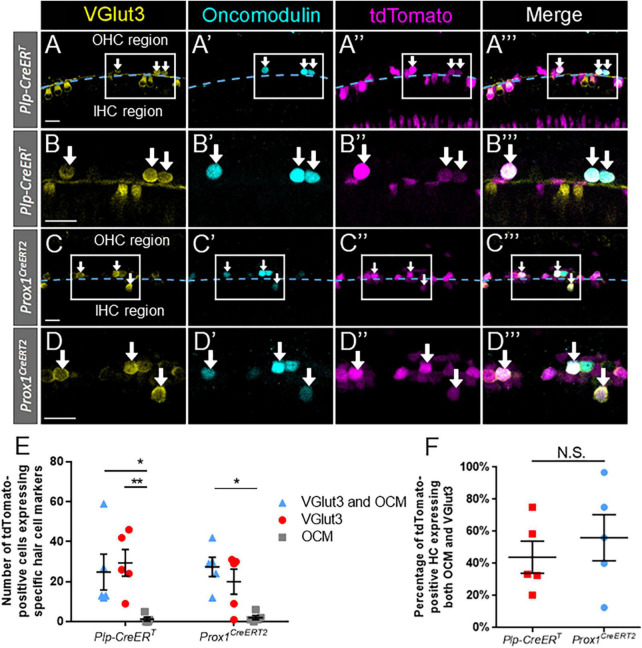
A large proportion of regenerated hair cells expressed both oncomodulin and VGlut3 across supporting cell subtypes. **(A–C”’)** Representative confocal slice images from the apical turn of *Plp-CreER*^T^*:Rosa26*^tdTomato^*:Pou4f3*^DTR^** **(A–B”’)** and *Prox1*^CreERT2^*:Rosa26*^tdTomato^*:Pou4f3*^DTR^** **(C–D”’)** mice at P7–P8 that were injected with tamoxifen at P0 to induce tdTomato expression in the two supporting cell subpopulations and with DT at P1 to induce hair cell damage. Samples were immunostained with anti-VGlut3 antibodies (yellow) as a marker of mature inner hair cells and anti-oncomodulin antibodies (blue) as a marker of immature outer hair cells. tdTomato was detected using endogenous fluorescence (magenta). Arrows indicate VGlut3-positive/oncomodulin-positive/tdTomato-positive cells that are regenerated, fate-mapped hair cells. **(A–A”’,C–C”’)** Based on their location relative to the tunnel of Corti (dashed blue line), regenerated hair cells were classified as being located in the lateral compartment (upper half of image) or the medial compartment (lower half of image) position. Boxes outline the location where higher magnification images **(B–B”’, D–D”’)** were taken. All scale bars: 25 μm. **(E)** In both population of supporting cells, a significantly greater number of regenerated hair cells (Tomato-positive) expressed both VGlut3 and oncomodulin (blue triangles) compared to those expressing only oncomodulin (gray squares). For regenerated hair cells fate-mapped by *Plp-CreER*^T^**, the number of cells expressing only VGlut3 (red circles) also significantly exceeded those expressing only oncomodulin. **(F)** When normalized to the total number of tdTomato- positive hair cells, there was no significant difference in the percentage of VGlut3-positive/oncomodulin-positive regenerated hair cells between the supporting cell populations targeted by the two CreER lines. *p < 0.05; **p < 0.01, determined using two-way ANOVA with a Sidak’s *post-hoc* test. Data are presented as mean ± SEM; *n* = 5. N.S., not significant.

We observed a significant number of regenerated hair cells (tdTomato-positive) that expressed VGlut3 without oncomodulin in both lines (*DTR:Prox1^CreERT2^*: 20.0 ± 6.3; *DTR:Plp-CreER*^T^**: 29.4 ± 6.7, *n* = 5) (Figures 6A–E). Conversely, an exceedingly low number of oncomodulin-positive regenerated hair cells (tdTomato-positive) expressed oncomodulin without VGlut3 (*DTR:Prox1^CreERT2^*: 2.0 ± 1.0; *DTR:Plp-CreER*^T^**: 1.2 ± 1.0, *n* = 5) (Figures 6A–E). Within the *DTR:Prox1^CreERT2^* cochleae, there was a significantly greater number of regenerated hair cells expressing both VGlut3 and oncomodulin than those expressing oncomodulin alone (27.4 ± 4.9 vs. 2.0 ± 1.0, *n* = 5, *p* = 0.0118, determined using two-way ANOVA followed by a Sidak’s *post-hoc* test) (Figures 6A–E). Within the *DTR:Plp-CreER*^T^** cochleae, there was a significantly greater number of regenerated hair cells expressing VGlut3 alone or both VGlut3 and oncomodulin than those expressing oncomodulin alone (24.8 ± 9.0 vs. 1.2 ± 1.0, *n* = 5, *p* = 0.0050 and 29.4 ± 6.7 vs. 1.2 ± 1.0, *n* = 5, *p* = 0.0201, determined using two-way ANOVA followed by a Sidak’s *post-hoc* test) (Figures 6A–E). When the data were normalized to the total number of regenerated hair cells (tdTomato-positive cells that co-expressed either VGlut3 or oncomodulin), a similar percentage of cells expressing both oncomodulin and VGlut3 were seen in both CreER lines (*Prox1^CreERT2^*: 59.3 ± 12.2%; *DTR:Plp-CreER*^T^**: 43.9 ± 9.9%, *n* = 5, determined using an unpaired Student’s *t*-test) (Figure 6F).

Since *Plp-CreER*^T^** is expressed in ∼7–11% of inner pillar, outer pillar, and Deiters’ cells in addition to the supporting cells in the medial compartment (Figures 1B,D,D’; McGovern et al., 2017), we closely examined the location of the regenerated hair cells, using the tunnel of Corti as a landmark (Figures 6A–A”’,C–C”’). Regenerated hair cells that expressed both oncomodulin and VGlut3 simultaneously were most commonly found within the lateral compartment (*Prox1^CreERT2^*: 23.8 ± 4.0; *Plp-CreER*^T^**: 21.6 ± 9.8, *n* = 5), and were rarely seen in the medial compartment (*Prox1^CreERT2^*: 3.6 ± 1.4; *Plp-CreER*^T^**: 3.2 ± 1.0, *n* = 5) in both CreER lines (Figures 6B–B”’,D–D”’). While the regenerated hair cells that expressed VGlut3 without oncomodulin were found exclusively medial to the tunnel of Corti, the cells that expressed oncomodulin without VGlut3 were almost exclusively observed in the lateral compartment. When the data were normalized to the total number of regenerated hair cells (tdTomato-positive cells that co-expressed either VGlut3 or oncomodulin), a similar percentage of cells expressing both oncomodulin and VGlut3 were seen in the lateral compartment of both CreER lines (*Prox1^CreERT2^*: 87.7 ± 3.8%; *DTR:Plp-CreER*^T^**: 77.0 ± 9.0%, *n* = 5, determined using an unpaired Student’s *t*-test). This suggests that VGlut3 expression is not properly controlled in the neonatal cochlea, thus many regenerated hair cells may develop as hybrids, expressing markers of both inner and outer hair cells.

### Regenerated hair cells from both mouse lines form new synaptic connections

**FIGURE 7:**
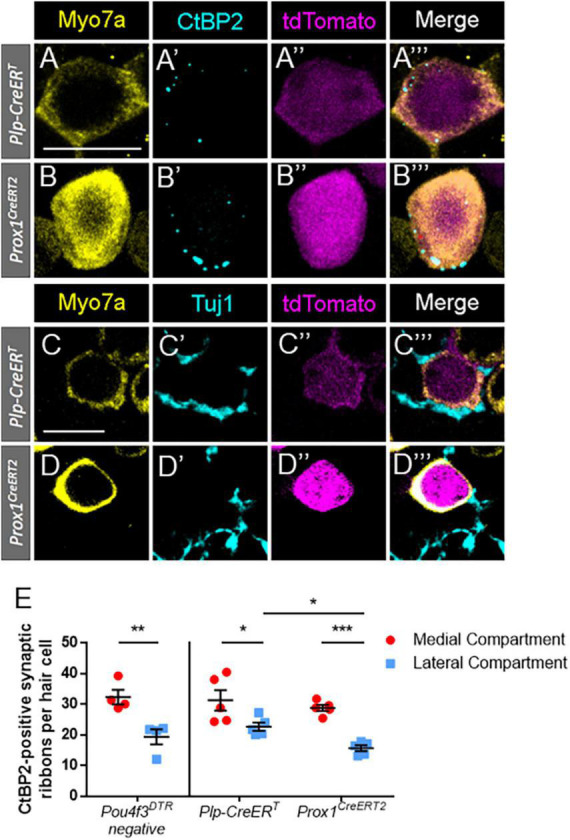
Regenerated hair cells formed ribbon synapses and were innervated by Tuj1-positive neurites at P7. **(A–D”’)** Representative confocal slice images from the apical turn of *Plp-CreER*^T^*:Rosa26*^tdTomato^*:Pou4f3*^DTR^** **(A–A”’,C–C”’)** and *Prox1*^CreERT2^*:Rosa26*^tdTomato^*:Pou4f3*^DTR^** **(B–B”’,D–D”’)** mice at P7–P8 that were injected with tamoxifen at P0 to induce tdTomato expression in the two supporting cell subpopulations and with DT at P1 to induce hair cell damage. Samples were immunostained with anti-Myo7a antibodies (yellow) to label hair cells and anti-CtBP2 antibodies (blue, **A–B”’**) to label synaptic ribbons or anti-Tuj1 antibodies (blue, **C–D”’**) to label neurites of the spiral ganglion. tdTomato was detected using endogenous fluorescence (magenta). Scale bars: 10 μm. **(E)** In the *Pou4f3^DTR^*-negative controls, there were significant more ribbon synapses in the inner hair cells located in the medial compartment (red circles) than the outer hair cells located the in the lateral compartment (blue squares). For the *DTR:Prox1*^CreERT2^** and *DTR:Plp-CreER*^T^** mice, hair cells were classified as located in the medial or lateral compartment based on their location relative to the tunnels of Corti (see Figures 6A–A”’,C–C”’). Similar to the control dataset, significantly more synaptic ribbons were seen in regenerated hair cells (Myo7a-poitive/Tomato-positive) located in the medial compartment compared to the lateral compartment for both CreER lines. In addition, there were a greater number of CtBP2-positive puncta in the lateral compartment regenerated hair cells fate-mapped by *Plp-CreER*^T^** than those fate-mapped by *Prox1^CreERT2^*. *p < 0.05; **p < 0.01; ***p < 0.001, determined using an unpaired Student’s *t*-test. Data are presented as mean ± SEM; *n* = 4–5.

Hair cell types also differ in their innervation patterns with afferent spiral ganglion neurons. To identify synapses and innervation with regenerated hair cells, cochlear sections of both CreER lines at P7 were stained with either anti-C-terminal binding protein 2 (CtBP2) (Figures 7A–B”’) or anti-neuron-specific class III β-tubulin (Tuj1) (Figures 7C–D”’). CtBP2 is an important component of the ribbon synapses that are found in both inner and outer hair cells (Schmitz, 2009; Uthaiah and Hudspeth, 2010; Becker et al., 2018). Tuj1 is an important structural component of spiral ganglion neurites (Sekerková et al., 2008). In the *DTR:Plp-CreER*^T^**- and *DTR:Prox1^CreERT2^*-labeled regenerated hair cells (tdTomato-positive/Myo7a-positive) at P7, 81.8 and 96.8% had numerous CtBP2-positive puncta, respectively, while 95.2 and 98.0% of regenerated hair cells had direct contact with at least one Tuj1-positive neurite.

We next categorized tdTomato-positive hair cells as being in the medial or lateral compartments depending on their position relative to the tunnel of Corti (Figures 6A–A”’,C–C”’). At P7, each *DTR:Plp-CreER*^T^** cochleae contained 16.6 ± 4.2 medially positioned and 4.6 ± 0.9 laterally positioned tdTomato-positive hair cells, contrasting *DTR:Prox1^CreERT2^* cochleae, which each contained 4.0 ± 0.8 medially positioned cells and 77.8 ± 8.0 laterally positioned cells. Using the 10 most apical regenerated hair cells (tdTomato-positive/Myo7a-positive) in each compartment, we quantified the number of synapses per regenerated hair cell by counting CtBP2-positive puncta. In some cases there were less than 10 regenerated hair cells in a compartment, so we used all cells present in that sample/compartment for the quantifications. Medial compartment tdTomato-positive hair cells had significantly more CtBP2-positive ribbon synapses than lateral compartment tdTomato-positive cells in both the *DTR:Plp-CreER*^T^** and *DTR:Prox1^CreERT2^* lines (Figure 7E; 31.2 ± 3.4 vs. 22.6 ± 1.3 for *DTR:Plp-CreER*^T^**, *n* = 5, *p* = 0.0128 and 28.8 ± 1.0 vs. 15.6 ± 0.9 for *DTR:Prox1^CreERT2^*, *n* = 5, *p* = 0.0004 determined using two-way ANOVA followed by a Sidak’s *post-hoc* test). Furthermore, lateral compartment regenerated hair cells fate-mapped by *DTR:Plp-CreER^T^* had more CtBP2-positive puncta per cell than those fate-mapped by *DTR:Prox1^CreERT2^* (22.6 ± 1.3 vs. 15.6 ± 0.9, *n* = 5, *p* = 0.415 determined using two-way ANOVA followed by a Sidak’s *post-hoc* test). In P7 control samples (*Pou4f3*^DTR^*-negative*), there were also more ribbon synapses in inner hair cells (located in the medial compartment) compared to outer hair cells (located in the lateral compartment) (32.3 ± 2.36 vs. 19.3 ± 2.44, *N* = 4, *p* = 0.0086 determined using a Student’s *t*-test). However, there was no differences in the number of ribbon synapses per hair cell between controls and either CreER line for either compartment (Figure 7E). These synapse numbers are similar to the number of CtBP2-positive synaptic ribbons reported previously at P0-P3 during normal cochlear development (Huang et al., 2012), but greater than what is present in adult mice (Thiers et al., 2008; Becker et al., 2018; Chen et al., 2021), suggesting that at P7 hair cells have yet to undergone synaptic pruning.

## Discussion

While hair cells expressing markers of terminal differentiation have been reported in models where supporting cells were reprogrammed to induce hair cell regeneration (Liu et al., 2014; Lee et al., 2020; Chen et al., 2021), examples of this in spontaneously regenerated hair cells is limited. Previous work showed that some spontaneously regenerated hair cells can express the terminal outer hair cell marker prestin, but there is no evidence of inner hair cell replacement (Bramhall et al., 2014; Cox et al., 2014). Our work here, which includes cells that were regenerated by both mitotic regeneration and direct transdifferentiation, demonstrates that spontaneously regenerated hair cells are able to express markers of inner or outer hair cells, including their terminal differentiation markers, VGlut3 and prestin, respectively. In addition, a large number of supporting cells responded to hair cell damage by expressing Atoh1, within 2 days of the injury, yet the number of these cells that progressed to express Myo7a was significantly reduced in both CreER lines. The lack of progression toward a hair cell fate may be explained by recent findings which suggest that the targets of Atoh1 are not accessible in many neonatal supporting cells, or there may be a critical level of *Atoh1* expression needed to induce transdifferentiation in a feed-forward fashion through expression of *Pou4f3* (Yu et al., 2021).

### VGlut3 is mis-expressed in regenerated hair cells originating from supporting cells in the lateral compartment

Interestingly, when we co-stained for VGlut3 and oncomodulin simultaneously, we observed that the majority of the oncomodulin-positive, VGlut3-positive regenerated cells were found in the lateral compartment (87.7 ± 3.8% for *DTR:Prox1^CreERT2^* and 77.0 ± 9.0% for *DTR:Plp-CreER*^T^**, *N* = 5) While very few regenerated cells expressed oncomodulin alone, we found that about a quarter of regenerated cells expressed VGlut3 alone in each CreER line, with these cells located exclusively in the medial compartment.

The finding of these outer compartment hybrid cells suggests that the cues that control regulation of inner hair cell genes and differentiation are reduced or are no longer present in the neonatal cochlea. *Atoh1* and *Pou4f3* have been shown to be important for induction of a general cochlear hair cell fate. Two additional transcription factors, Ikaros family zinc finger 2 (*Ikzf2*) (which encodes the protein Helios) and insulinoma-associated protein 1 (*Insm1*) have recently been implicated in further differentiation toward an outer hair cell fate (Chessum et al., 2018; Wiwatpanit et al., 2018; Sun et al., 2021) and *Tbx2* has been implicated in promoting an inner hair cell fate (García-Añoveros et al., 2022). *Ikzf2* is expressed starting at P4, and persists in fully mature outer hair cells. It is thought to play a role in promoting an outer hair cell fate by downregulating genes that are inner hair cell-specific, and upregulating outer hair cell-specific genes (Chessum et al., 2018). Conversely, *Insm1* is expressed only transiently from embryonic day (E) 15.5 to P2 in outer hair cells (Lorenzen et al., 2015). It is also thought to help promote an outer hair cell fate by repressing inner hair cell-specific genes and preventing embryonic hair cells from responding to pro-inner hair cell signaling gradients. Deletion of *Insm1* during embryonic development caused differentiation of some hair cells in the lateral compartment into inner hair cells in a decreasing gradient from the medial to lateral compartments (Wiwatpanit et al., 2018). The recently described opposing medial-lateral gradients of Activin A and Follistatin may represent two such gradients driving this process (Prajapati-DiNubila et al., 2019). Given that the induction of hair cell damage in our work occurred at P1, which is near the end of normal *Insm1* expression, it is possible that spontaneously regenerating hair cells in the lateral compartment lacked appropriate expression of *Insm1* and were thus susceptible to molecular gradients driving an inner hair cell fate, leading to the inappropriate expression of VGlut3 that we observed. Another potential explanation is that the hybrid cells (VGlut3-positive/oncomodulin-positive/Tomato-positive) are original hair cells that survived the DT-mediated damage. However this is unlikely since there was only ∼1 Tomato-positive hair cell in *Plp-CreER^T^* mice and ∼13 Tomato-positive hair cells in *Prox1^CreERT2^* mice and 30–40 hybrid cells present in both mouse models.

It is unclear what properties these hybrid cells might possess. It is also possible that the VGlut3 mis-expression induced cell death, since previous reports showed that most regenerated hair cells do not survive into adulthood (Cox et al., 2014). Although examples of cell transdifferentiation are seen in other organ systems, such as the liver and pancreas (Shen et al., 2003; Yanger et al., 2013), to the best of our knowledge similar evidence of healthy, regenerated cells co-expressing markers of other distinct, neighboring cell types has not been reported. Rather, inappropriate expression of proteins is often discussed within the context of dysplasia or malignancy (Qie and Diehl, 2016; Kent and Leone, 2019).

### Regenerated hair cells express mature inner and outer hair cell markers, and the type of marker expressed does not depend on the supporting cell subtype of origin

A recent single cell RNA-seq study showed that the inner phalangeal cells located in the medial compartment of the organ of Corti have a very different molecular profile than the pillar and Deiters’ cells located in the lateral compartment (Kolla et al., 2020). We attempted to determine whether supporting cells in closest proximity to each hair cell subtype would be more likely to produce the same type of regenerated hair cell (i.e., are the supporting cells that neighbor each hair cell type pre-programmed to follow a specific fate). Contrary to our hypothesis, we found that there were no differences in the percentage of regenerated hair cells from the two CreER lines that expressed markers of an immature outer hair cell fate (by expressing oncomodulin) or a mature inner hair cell fate (by expressing VGlut3). Conversely, there was a greater proportion of *Prox1^CreERT2^*–labeled regenerated cells that expressed the terminal outer hair cell gene, prestin. Importantly, these experiments were performed with only one type-specific hair cell marker, along with the general hair cell marker, Myo7a. While this allowed us to characterize differentiated cells as a proportion of all regenerated cells labeled by a specific CreER line, our subsequent finding of hybrid cells suggests that experiments performed with only one type-specific marker did not reliably define a pure inner or outer hair cell phenotype. While our single-marker findings contradict with a recent report by Chen et al. (2021) in which hair cells induced by over-expression of *Gfi1*, *Pou4f3*, and *Atoh1* produced cells expressing prestin only when pillar and Deiters’ cells were targeted, and produced cells expressing VGlut3 only when inner phalangeal and border cells were targeted, these studies examined VGlut3 and prestin separately. In addition, the different results from these two studies may have been caused by changes that occurred in the organ of Corti after DT-induced hair cell damage, which was present in our experiment, but not in the Chen et al. (2021) study, or were due to the age of the supporting cells being studied.

### Plasticity differences between supporting cell subtypes in the medial and lateral compartments

While there was no difference in the percentage of regenerated hair cells (Myo7a-positive/Tomato-positive cells) from the two CreER lines, there were differences in the percentage of supporting cells from each population that turned on the early hair cell gene, Atoh1, after hair cell damage. Specifically, there were ∼2 fold more *Plp-CreER*^T^**-positive supporting cells that expressed Atoh1 after damage and this may suggest that supporting cells in the medial compartment, located near inner hair cells, retain their plasticity and capacity to respond to hair cell damage longer than other supporting cell subtypes. When looking at cochlear development, this may seem surprising since the hair cells and supporting cells in the medial compartment differentiate from progenitor cells before the cells in the lateral compartment (Morrison et al., 1999; Lanford et al., 2000; Driver et al., 2013), and thus one might predict that the medial cells are more mature at neonatal ages.

It is well documented that the supporting cells in the base of the cochlea begin the maturation process 1–2 days before the cells in the apex (Hallworth et al., 2000; Jensen-Smith et al., 2003; Szarama et al., 2012) and that the majority of regenerated hair cells are located in the apical turn of the cochlea where supporting cells are less mature (Bramhall et al., 2014; Cox et al., 2014; Atkinson et al., 2018; McGovern et al., 2019), Similar to previous reports (Atkinson et al., 2018), the *Prox1^CreERT2^*–labeled supporting cells in the lateral compartment followed this gradient showing a decline in the number of Atoh1-positive cells from the apex to the base. However there was no gradient of Atoh1 expression seen for the *Plp-CreER*^T^**-labeled supporting cells, which also supports the idea that medial supporting cells remain plastic for a longer period of time.

### Spontaneously regenerated hair cells form new synaptic connections

Contact with Tuj1-positive neurites and formation of CtBP2-positive synaptic boutons was seen in the vast majority of regenerated hair cells, suggesting that the replacement hair cells form new synaptic connections. During normal embryogenesis and the early postnatal period, the number of synaptic ribbons in each hair cell increases gradually. Inner hair cells reach a peak of 50–55 CtBP2-positive puncta around P3, whereas outer hair cells reach a peak of 45–50 CtBP2-positive puncta around P6. Both subsets of hair cells are then subject to synaptic pruning until ∼P12, at which time they reach adult levels (Huang et al., 2012). In adult mice, the number of CtBP2 puncta has been reported to be approximately 15–20 per inner hair cell (Thiers et al., 2008; Becker et al., 2018; Chen et al., 2021) and 1–2 per outer hair cell (Thiers et al., 2008). The number of CtBP2-positive synaptic ribbons seen in regenerated hair cells in our experiments at P7 are similar to the numbers of ribbon synapses seen in the first few postnatal days during normal development, based on data from both with our control samples and published data. This is logical since the regenerated cells had just differentiated from supporting cells a few days prior, and suggests that the synaptic pruning process has not yet occurred. Additionally, lateral compartment regenerated cells contained significantly less synapses than the medial compartment cells, which follows the normal pattern of synapse counts seen in normal inner and outer hair cells.

In summary our study showed that regenerated hair cells expressing terminal differentiation markers of both inner and outer hair cells could be produced from either subset of fate-mapped supporting cells, and that these regenerated cells contained synaptic boutons and neuronal connections. Our work also suggests that the regulatory factors guiding terminal hair cell differentiation are altered during the first week of neonatal life, as evidenced by aberrant expression of VGlut3 in regenerating cells located in the lateral, outer hair cell compartment. Further work is needed to elucidate the processes that guide hair cell differentiation into subtypes, as replicating these processes during regeneration will likely be necessary to form functional, mature inner and outer hair cells.

## Materials and methods

### Animals

*Plp-CreER*^T^** mice (stock #5975, Doerflinger et al., 2003), *Rosa26^tdTomato^* mice, also referred to as Ai14 (stock #7914, Madisen et al., 2010), and *Atoh1^GFP/+^* mice (stock #13593, Rose et al., 2009) were obtained from The Jackson Laboratory. *Pou4f3^DTR^* mice (Golub et al., 2012; Tong et al., 2015) were provided by Dr. Ed Rubel (University of Washington, Seattle, WA, USA). *Prox1^CreERT2^* mice (Srinivasan et al., 2007) were provided by Dr. Guillermo Oliver (St. Jude Children’s Research Hospital, Memphis, TN, USA). Both of these strains are now available at The Jackson Laboratory. Transnetyx, Inc., performed all genotyping. Mice of both genders were used, and all animal work was performed in accordance with approved animal protocols from the Institutional Animal Care and Use Committee at Southern Illinois University School of Medicine.

### Substances given to animals

tdTomato expression was induced in supporting cell subgroups by intraperitoneal injection of 3 mg/40 g tamoxifen (Sigma-Aldrich, St. Louis, MO, USA) in 100% corn oil (Sigma-Aldrich, St Louis, MO, USA) given at P0. Hair cell death was induced by intramuscular injection of 6.25 ng/g DT (List Biological Laboratories, Inc., Campbell, CA, USA) given at P1.

### Immunostaining

Neonatal pups were euthanized under isoflurane anesthesia (Piramal Enterprises Limited, Telangana, India) between P3-P10 and cochleae were subsequently harvested and post-fixed in 4% paraformaldehyde (Polysciences, Inc., Warrington, PA, USA) for ∼2 h at room temperature. Samples were then transferred to 10 mM phosphate buffered saline (PBS) (Sigma-Aldrich, St. Louis, MO, USA) and stored at 4°C. Whole mount dissection was performed with cochleae dissected into 3 turns of equal length. Sections were placed in a 48-well plate for free floating immunostaining as previously described (Montgomery and Cox, 2016). The following primary antibodies were used: rabbit anti-myosin VIIa (Myo7a) (1:200, cat#25-6790, Proteus Biosciences, Ramona, CA, USA), goat anti-prestin (1:200, cat#sc-22692, Santa Cruz Biotechnology, Inc., Dallas, TX, USA), mouse anti-CtBP2 IgG1 (1:500, cat#BDB612044, Fisher, Hampton, NH, USA), mouse anti-Tuj1 IgG2a (1:500, cat#801201, BioLegend, San Diego, CA, USA), chicken anti-GFP (1:1,000, cat#ab13970, Abcam, Cambridge, United Kingdom), goat anti-oncomodulin (1:200, cat#sc-7446, Santa Cruz Biotechnology, Inc., Dallas, TX, USA), mouse anti-VGlut3 IgG2a (1:200, cat#135211, Synaptic Systems, Göttingen, Germany), and rabbit anti-VGlut3 (1:500, cat#135203, Synaptic Systems, Göttingen, Germany). Alexa fluor-conjugated secondary antibodies (Invitrogen, Thermo Fisher Scientific, Carlsbad, CA, USA) were used at 1:1000 dilution and nuclei were stained with Hoechst (1:2000, Fisher, Hampton, NH, USA). Samples were mounted on slides using Prolong Gold (Fisher, Hampton, NH, USA).

### Cell counts

Confocal imaging was performed using the Zeiss LSM800 (Zeiss, Oberkochen, Germany), and images were processed using Zen Blue (Zeiss, Oberkochen, Germany) software. All Myo7a-positive/tdTomato-positive cells (regenerated hair cells) were quantified and assessed for expression of the various markers along the entire length of the cochlea for both experimental and control groups. Regenerated hair cells that expressed specific markers of inner or outer hair cells were normalized to the total number of Myo7a-positive, tdTomato-positive cells in each cochlea. In Figure 2, all tdTomato-positive supporting cells throughout the cochlea that expressed ATOH1-GFP or Myo7a were included in the quantifications. In Figure 2E, total numbers of Tomato-positive supporting cells in each CreER line were taken from McGovern et al. (2019) to generate the percentages. In Figure 5, regenerated hair cells were identified by tdTomato expression combined with expression of either or both oncomodulin and VGlut3 (since Myo7a was not used in this experiment). For quantification of CtBP2-positive synapses in Figure 7, up to 10 regenerated hair cells (Myo7a-positive, tdTomato-positive) in each compartment (medial and lateral) were assessed in each cochlea. When more than 10 cells in each compartment were present in a single cochlea, the 10 most apical cells were used for synapse quantification.

### Statistical analysis

All data are presented as mean ± SEM and the *n*-value represents the number of mice included in the study in which one cochlea from each mouse was used for each experiment. Statistical tests were performed using GraphPad Prism 6.02 and are described in the figure legends.

## Data availability statement

The original contributions presented in this study are included in the article/supplementary material, further inquiries can be directed to the corresponding author.

## Ethics statement

The animal study was reviewed and approved by Institutional Animal Care and Use Committee at Southern Illinois University School of Medicine.

## Author contributions

MH performed the experiments and data analysis, as well as contributed to the experimental design under the supervision of BC. DH and SM performed the experiments and edited the manuscript. BC conceived the project idea and together with MH wrote and edited the manuscript. All authors contributed to the article and approved the submitted version.
